# Mastery Is Associated With Weight Status, Food Intake, Snacking, and Eating Disorder Symptoms in the NutriNet-Santé Cohort Study

**DOI:** 10.3389/fnut.2022.871669

**Published:** 2022-05-25

**Authors:** Ulrike A. Gisch, Margaux Robert, Noémi Berlin, Antoine Nebout, Fabrice Etilé, Sabrina Teyssier, Valentina A. Andreeva, Serge Hercberg, Mathilde Touvier, Sandrine Péneau

**Affiliations:** ^1^Sorbonne Paris Nord University, Inserm U1153, Inrae U1125, Cnam, Nutritional Epidemiology Research Team (EREN), Centre of Research in Epidemiology and Statistics - University of Paris (CRESS), Bobigny, France; ^2^Counseling Psychology, Department of Psychology, University of Potsdam, Potsdam, Germany; ^3^CNRS, EconomiX – UMR 7235, University of Paris Nanterre, Ivry-sur-Seine, France; ^4^INRAE, UR 1303 ALISS, Ivry-sur-Seine, France; ^5^Paris School of Economics and INRAE, UMR1393 PjSE, Paris, France; ^6^Grenoble Alpes University, INRAE, CNRS, Grenoble INP, GAEL, Grenoble, France; ^7^Department of Public Health, AP-HP Avicenne Hospital, Bobigny, France

**Keywords:** mastery, locus of control, weight status, diet quality, food group consumption, snacking, eating disorder symptoms, large population

## Abstract

Mastery is a psychological resource that is defined as the extent to which individuals perceive having control over important circumstances of their lives. Although mastery has been associated with various physical and psychological health outcomes, studies assessing its relationship with weight status and dietary behavior are lacking. The aim of this cross-sectional study was to assess the relationship between mastery and weight status, food intake, snacking, and eating disorder (ED) symptoms in the NutriNet-Santé cohort study. Mastery was measured with the Pearlin Mastery Scale (PMS) in 32,588 adults (77.45% female), the mean age was 50.04 (14.53) years. Height and weight were self-reported. Overall diet quality and food group consumption were evaluated with ≥3 self-reported 24-h dietary records (range: 3–27). Snacking was assessed with an *ad-hoc* question. ED symptoms were assessed with the Sick-Control-One-Fat-Food Questionnaire (SCOFF). Linear and logistic regression analyses were conducted to assess the relationship between mastery and weight status, food intake, snacking, and ED symptoms, controlling for sociodemographic and lifestyle characteristics. Females with a higher level of mastery were less likely to be underweight (OR: 0.88; 95%CI: 0.84, 0.93), overweight [OR: 0.94 (0.91, 0.97)], or obese [class I: OR: 0.86 (0.82, 0.90); class II: OR: 0.76 (0.71, 0.82); class III: OR: 0.77 (0.69, 0.86)]. Males with a higher level of mastery were less likely to be obese [class III: OR: 0.75 (0.57, 0.99)]. Mastery was associated with better diet quality overall, a higher consumption of fruit and vegetables, seafood, wholegrain foods, legumes, non-salted oleaginous fruits, and alcoholic beverages and with a lower consumption of meat and poultry, dairy products, sugary and fatty products, milk-based desserts, and sweetened beverages. Mastery was also associated with lower snacking frequency [OR: 0.89 (0.86, 0.91)] and less ED symptoms [OR: 0.73 (0.71, 0.75)]. As mastery was associated with favorable dietary behavior and weight status, targeting mastery might be a promising approach in promoting healthy behaviors.

**Clinical Trial Registry Number:**

NCT03335644 at Clinicaltrials.gov.

## Introduction

Psychological factors are linked to overweight and obesity (1), eating disorders (ED) (2), and dietary intake (3, 4). In recent decades, research on psychological factors has shifted from assessing negative and pathological factors to positive factors (5). Positive factors can be easily targeted in interventions [with e.g., goal setting or using personal strengths (6)] and represent important avenues to foster health over and above the absence of illness (7).

Mastery is defined as the extent to which individuals perceive having control over important circumstances of their lives (8). It is a psychological resource that helps individuals to cope with life events and life strains (9). Mastery is not regarded as a stable personality trait but as an adaptive self-concept that changes with critical experiences (10). As mastery is considered a modifiable factor (11), it may be a potential facilitator in promoting healthy dietary behavior.

Previous research has shown that mastery is associated with various physical health outcomes including better cardiometabolic health (10, 12, 13) or reduced mortality risk (12, 14) as well as psychological health outcomes including higher self-esteem (13, 15), sense of coherence (13, 16), life satisfaction (16) and lower depression (15, 17). However, research on the relationships between mastery and weight status as well as dietary behavior remain scarce and inconsistent. With regard to the relationship between mastery and weight status, different studies have found a negative relationship in both males and females (10, 15), a positive relationship in male students (18) and no relationship in females (18–20) or males (19). Only a few studies investigated the relationship between mastery and food intake and revealed inconsistent results. Mastery was positively associated with the Healthy Eating Index and several of its indicators (21) in a large population. However, other studies showed no association between mastery and diet quality (22), fat or fiber intake (23) while there were inconsistent results regarding fruit and vegetable consumption (24). To our knowledge, no study has investigated the relationship between mastery and snacking. As frequent snacking can be seen as a maladaptive coping behavior to deal with stressors (25, 26), it is conceivable that individuals who snack frequently, unlike those who do not snack frequently, show lower levels of mastery. Previous results regarding the relationship between mastery and EDs revealed a negative association between mastery and the overall level of eating pathology (20), binge eating (19) as well as weight concern, shape concern and eating concern (27). However, females with and without an ED reported a similar level of mastery (20).

To better understand the role of mastery with regard to dietary behavior, more research is needed, especially in a large population. Many studies on mastery have been conducted in specific populations, e.g., students, clinical populations or older adults, and do not consider potential confounders. Therefore, the aim of the current study was to investigate the association between mastery and weight status, diet quality, food group consumption, snacking and ED symptoms in a large sample of French adults, taking into account sociodemographic and lifestyle characteristics.

## Materials and Methods

### Population and Study Design

To conduct this study, we used data of the NutriNet-Santé study. The NutriNet-Santé study was launched in France in 2009 and is a large ongoing web-based prospective cohort study in the French population (https://etude-nutrinet-sante.fr/). It aims at examining the relationship between nutrition and health as well as investigating determinants of dietary patterns and nutritional status in adults aged ≥18 years (28). At inclusion, participants were asked to fill out web-based questionnaires assessing diet, physical activity, anthropometric measures, lifestyle characteristics, socioeconomic conditions, and health status. Participants were asked to fill out this set of questionnaires every year after inclusion. Furthermore, participants were asked every month to complete another set of optional questionnaires assessing determinants of eating behavior, nutritional status and specific health-related aspects. Please see the study protocol for further information regarding the methodology and design (28).

### Instruments

#### Mastery

Mastery was assessed with a translated French version of the Pearlin Mastery Scale (PMS) (9). The PMS was once administered between May and November 2014. Responses to its 7 items were recorded on a 7-point scale ranging from 1 (*totally disagree*) to 7 (*totally agree*). Item scores were summed and then divided by the number of items, leading to a score ranging from 1 to 7. Higher mean values reflect higher levels of mastery. Previous studies have found support for the scale's validity and reliability (16, 29).

#### Weight Status

BMI was assessed with self-reported height and weight. BMI (in kg/m^2^) was calculated as the ratio of weight to squared height. Anthropometric data provided closest after completion of the PMS were used. Participants were categorized as underweight (BMI <18.5 kg/m^2^), normal weight (18.5 kg/m^2^ ≤ BMI <25 kg/m^2^), overweight (excluding obesity) (25 kg/m^2^ ≤ BMI <30 kg/m^2^), obese class I (30 kg/m^2^ ≤ BMI <35 kg/m^2^), obese class II (35 kg/m^2^ ≤ BMI <40 kg/m^2^), and obese class III (BMI ≥ 40 kg/m^2^) (30).

#### Diet Quality and Food Group Consumption

At inclusion and every 6 months afterwards, participants were asked to complete a set of three 24-h dietary records (randomly distributed between 2 weekdays and 1 weekend day). Individuals who completed ≥3 24-h dietary records in the time-frame of 2 years before and 2 years after the completion of the PMS were selected for the present study. The number of completed 24-h records ranged from 3 to 27. An interactive web-based interface allowed the participants to complete the dietary record on their own by choosing among >3,500 food or beverage items (31). Participants were asked to report all foods and beverages consumed at mealtimes (breakfast, lunch, dinner) and other eating occasions. They estimated the amounts consumed using standard measurements or validated photographs (32). To assess portion sizes, participants had to choose between 7 categories for most food products: 3 main portion sizes, 2 intermediate portion sizes and 2 extreme portion sizes. Based on the NutriNet-Santé food composition table (33), nutrient intakes were estimated. Mean daily food intake (grams/day) was weighted according to weekday vs. weekend. We used the method proposed by Black (34) to identify participants with unlikely estimates of energy intake as under-reporters. We calculated the basal metabolic rate according to age, sex, weight, and height by using Schofield's equations (35). Based on basal metabolic rate and the level of physical activity, we determined energy requirement. We calculated the ratio between energy intake and estimated energy requirement and excluded individuals with ratios below the Goldberg cutoff (36). The validity of the dietary records in the NutriNet-Santé study has been shown by comparing the dietary records with biomarkers (37, 38) and with interviews by a dietitian (31). For this study, we defined 19 food groups: fruit and vegetables, seafood (fish and shellfish), meat and poultry, processed meat, eggs, dairy products (e.g., milk, yogurts with ≤ 12% of added sugar), cheese, milk-based desserts (e.g., flan, milk shakes), starchy foods, wholegrain foods, legumes, fats (e.g., oil, butter), sugary and fatty foods (e.g., cakes, chocolate, ice cream, pancakes), sugar and confectionery (e.g., honey, jelly), fast food (e.g., pizzas, hamburgers, quiches), appetizers (e.g., chips, salted biscuits, salted oleaginous fruits), non-salted oleaginous fruits (e.g., non-salted nuts, non-salted almonds), sweetened beverages (sugary sweetened beverages and artificially sweetened beverages) and alcoholic beverages.

To assess diet quality, we used the modified French National Nutrition and Health Program Guideline Score (mPNNS-GS) which is an a priori diet quality score, reflecting the adherence to the French nutritional recommendations that were in effect at the time of the PMS measurement (39). It is based on the PNNS-GS score, however, it only accounts for the dietary component while excluding the physical activity component (39, 40). The mPNNS-GS includes 12 components: 8 components refer to food serving recommendations (fruit and vegetables; starchy foods; wholegrain foods; dairy products; meat, eggs, and fish; seafood; vegetable fat; water and soda) and 4 components refer to moderation of intake (added fat, salt, sweets, and alcohol). For overconsumption of salt, overconsumption of added sugars from sweetened foods and when energy intake exceeds energy requirement [based on the level of physical activity level and basal metabolic rate (35)] by > 5%, the score will be reduced. The maximum of the mPNNS-GS are 13.5 points, with higher scores indicating a better diet quality.

#### Snacking

Between April and October 2014, a meal pattern questionnaire was administered. Snacking was assessed with one question (“How often do you usually snack in the daytime?”). Responses were recorded on a 7-point scale ranging from *never* to *6 times or more per day, each day* and classified into 4 categories: never, < once a week, ≥ once a week (and < than once a day), or ≥ once a day. Further, we computed a binary variable: no snacking vs. snacking.

#### Eating Disorder (ED) Symptoms

ED symptoms were assessed with the French version of the SCOFF (Sick-Control-One-Fat-Food) questionnaire (41). The SCOFF was administered between June and December 2014. It includes 5 dichotomous items (yes vs. no) that cover the main features of EDs (42). A cut off ≥2 indicates ED symptoms (regardless of type). Previous studies support its reliability and validity and its suitability as a screening tool for EDs (43, 44). In the current sample, McDonald's omega (ω) as an index for reliability (45) was 0.550, 95% CI [0.540, 0.561]. Furthermore, we used the Expali algorithm that allows to distinguish between ED categories (46). Based on the answers given in the SCOFF and the individual's BMI, the Expali algorithm classified individuals with ED symptoms into 4 broad categories. These categories were based on the DSM-5 (47): (1) restrictive disorders including anorexia nervosa, restrictive food intake disorder and atypical anorexia nervosa; (2) bulimic disorders including bulimia nervosa and bulimia nervosa of low frequency or duration; (3) hyperphagic disorders including binge-eating disorder and binge-eating disorder of low frequency or duration; and 4. other ED including purging disorder, night eating syndrome and any other ED.

#### Covariates

Guided by previous studies, we collected data on potential confounders of the relationship between mastery, weight status, diet quality, food group consumption, snacking and EDs. Covariates were assessed at inclusion and once a year. We used the data provided closest to the date of the completion of the PMS. Selected covariates were as follows: age (years), sex, educational level (primary, secondary, undergraduate, and postgraduate), occupational status (unemployed, student, self-employed/farmer, employee/manual worker, intermediate profession, managerial staff/intellectual profession, and retired), monthly income per household unit (<1,200; 1,200–1,799; 1,800–2,299; 2,300–2,699; 2,700–3,699; ≥3,700 euros per household unit, and “unwilling to answer”), smoking status (never, former, and current smoker), level of physical activity (low, moderate, and high), and depressive symptomatology (yes, no). The covariates were determined as follows: we determined monthly income per household unit with information about income and household composition. According to the Organization for Economic Cooperation and Development equivalence scale, we converted the number of people in the household into number of consumption units: 1 consumption unit is attributed for the first adult in the household, 0.5 for other persons aged ≥14 years, and 0.3 for children aged <14 years (48). We determined physical activity with the short form of the French version of the International Physical Activity Questionnaire (49). We estimated energy expenditure expressed in metabolic equivalent of task minutes per week. With that, we categorized the 3 levels of physical activity [low (<30 min/d), moderate (30–60 min/d), and high (≥60 min/d)]. We assessed depressive symptomatology by using the French version of the Center for Epidemiological Studies–Depression (CES-D) scale (50, 51). Responses to its 20 items were recorded on a 4-point scale, with higher values indicating higher depressive symptomatology. A cut off ≥16 indicates a depressive symptomatology (51). In the current sample, McDonald's ω was 0.910, 95% CI [0.908, 0.912].

### Data Analysis

To compare the characteristics of included with excluded participants, we performed Student *t*-Tests for continuous variables and Pearson's chi-square tests for categorical variables. We assessed the reliability of the French version of the PMS by calculating McDonald's omega (ω) (45). To test the factor structure of the PMS, we performed a one factor confirmatory factor analysis (CFA). The following fit indexes with cut-off values for a good model fit were used to evaluate model fit: comparative fit index (CFI) ≥0.95, root mean square error of approximation (RMSEA) ≤ 0.06, and standardized root mean square residual (SRMR) ≤ 0.08 (52). To investigate the relationship between mastery and participants' characteristics, we used Spearman correlation coefficients (with 95% CI) for continuous variables and Student *t*-test or ANOVA for categorical variables. Depending on the variable level (categorical/continuous), we reported participants' characteristics and descriptive characteristics of outcomes either as percentages (%) or means (M) and standard deviations (SD). Medians and interquartile ranges (IQRs) were used for non-normally distributed intake of food groups. To investigate the relationship between mastery (independent variable, IV) and weight status (dependent variable, DV), we performed multinomial logistic regressions. Only these analyses were stratified by sex because the interaction between mastery and weight status was the only interaction between mastery and a DV that was significant (*p* = 0.005). To investigate the relationship between mastery (IV) and diet quality (DV) as well as normally distributed intake of food groups (DV) (fruit and vegetables, seafood, meat and poultry, dairy products, cheese, starchy foods, wholegrain foods, fats, sugary and fatty foods, sugar and confectionery), we performed multiple linear regressions. To investigate the relationship between mastery (IV) and non-normally distributed intake of food groups (DV) (processed meat, eggs, legumes, fast food, milk-based desserts, non-salted oleaginous fruits, appetizers, sweetened beverages, alcoholic beverages), we performed multinomial logistic regressions. For these groups, we defined three levels: no intake vs. low intake (< median intake among consumers) vs. high intake (≥median intake among consumers). To investigate the relationship between mastery (IV) and snacking (DV), we performed binary logistic regressions (snacking vs. no snacking) as well as multinomial logistic regressions with four frequency categories (never vs. < once a week vs. ≥ once a week vs. ≥ once a day). To investigate the relationship between mastery (IV) and ED symptoms (DV), we performed binary logistic regressions (ED symptoms vs. no ED symptoms) as well as multinomial logistic regressions with the four ED categories (no ED vs. restrictive disorder vs. bulimic disorder vs. hyperphagic disorder vs. other ED). From logistic regressions, we estimated the strength of the associations by the calculation of adjusted odds ratios (ORs) and 95% confidence intervals (95% CIs). All analyses were performed with (adjusted model) and without (unadjusted model) confounding variables. The adjusted model included age, sex, educational level, occupational status, monthly income per household unit, energy intake, smoking status, and physical activity. The analyses between mastery and food groups further included the number of 24-h dietary records as a confounding variable. Further, we computed a sensitivity analysis adding depressive symptomatology to the models. To handle missing data on covariates, we used multiple imputation by fully conditional specification (20 imputed data sets). All tests of statistical significance were 2-sided and significance was set at 5%. All statistical analyses were performed using SAS version 9.4 software (SAS Institute, Inc.), except for McDonald's ω that was calculated with the MBESS R package (version 4.8.0).

## Results

### Sample Characteristics

Of the 139,420 subjects who were included in the NutriNet-Santé study in 2014, 33,017 participants completed the PMS. We excluded 148 participants due to an acquiescence bias (i.e., agreeing or disagreeing with all statements without consideration of the reverse-worded items) and 281 participants who were pregnant, resulting in 32,588 participants eligible for analysis. Among those, 30,620 participants also completed the snacking assessment, 30,339 participants reported anthropometric data, 28,951 participants completed the SCOFF, 25,024 participants had available data to assess diet quality and 22,209 participants had data on food group intake (see Supplementary Figure 1). To make better use of available data, we performed each analysis on a different subsample. Compared with excluded participants of the NutriNet-Santé cohort who did not complete the PMS or were excluded due to an acquiescence bias or pregnancy, included participants were older, had a higher proportion of males, a higher educational level and income (all *p* < 0.001). Table 1 presents the individual characteristics of the overall sample and their relationships to level of mastery. On average, mastery was higher in males, in more educated participants, in self-employed/farmers and managerial staff/intellectual professions, in participants with a higher income, in smokers, in participants with a higher physical activity and in participants without depressive symptomatology. Mastery was negatively associated with age and positively associated with energy intake. Table 2 presents the descriptive statistics of the outcome variables.

**Table 1:** Individual characteristics of *N* = 32,588 participants (NutriNet-Santé cohort study, 2014).

	**% or Mean ±SD**	**PMS^a^**	***P* ^b^**
**All**		4.89 ± 1.14	
**Age (years)**	50.04 ± 14.53	−0.11 (−0.12, −0.10)	<0.0001
**Sex**			<0.0001
Female	77.45	4.85 ± 1.16	
Male	22.55	5.02 ± 1.06	
**Educational level**			<0.0001
Primary	2.05	4.50 ± 1.23	
Secondary	28.47	4.67 ± 1.18	
Undergraduate	31.88	4.89 ± 1.12	
Postgraduate	36.88	5.07 ± 1.08	
Missing data	0.71		
**Occupational status**			<0.0001
Unemployed	9.80	4.65 ± 1.26	
Student	2.88	5.04 ± 1.09	
Self-employed, farmer	1.91	5.22 ± 1.12	
Employee, manual worker	15.85	4.74 ± 1.15	
Intermediate professions	16.71	4.94 ± 1.07	
Managerial staff, intellectual profession	24.44	5.16 ± 1.03	
Retired	27.08	4.74 ± 1.16	
Missing data	1.33		
**Monthly household income**			<0.0001
<1,200 €	11.64	4.64 ± 1.25	
1,200–1,799 €	21.01	4.79 ± 1.15	
1,800–2,299 €	14.69	4.89 ± 1.11	
2,300–2,699 €	9.91	4.96 ± 1.09	
2,700–3,699 €	16.66	5.03 ± 1.08	
>3,700 €	12.13	5.17 ± 1.06	
Unwilling to answer	11.65	4.78 ± 1.15	
Missing data	2.31		
**Energy intake (kcal/d)**	1847.61 ± 497.12	0.04 (0.03, 0.05)	<0.0001
**Smoking**			0.0010
Never	49.95	4.87 ± 1.15	
Former	38.13	4.90 ± 1.13	
Current	11.90	4.93 ± 1.13	
Missing data	0.01		
**Physical activity**			<0.0001
Low	23.07	4.76 ± 1.16	
Moderate	42.15	4.91 ± 1.11	
High	34.57	4.95 ± 1.15	
Missing data	0.20		
**Depressive symptomatology (CES-D)^c^**			<0.0001
No (<16)	57.18	5.07 ± 1.03	
Yes (≥16)	18.28	4.15 ± 1.17	
Missing data	24.53		

a *PMS, Pearlin Mastery Scale, score from 1 to 7, higher values indicate higher mastery. PMS scores in Mean ± SD for categorical variables. Spearman correlation coefficients (with 95% CI) for continuous variables.*

b *P-values based on Spearman correlation coefficients for continuous variables and Student t-test or ANOVA for categorical variables.*

c *CES-D, Center for Epidemiological Studies Depression Scale*.

**Table 2:** Descriptive characteristics of the outcome variables (NutriNet-Santé cohort study, 2014).

	**% or Mean ±SD^a^**	**PMS^b^**	***P* ^c^**
**BMI^d^**			<0.0001
Underweight (<18.5 kg/m^2^)	4.64	4.81 ± 1.21	
Normal weight (18.5–25 kg/m^2^)	61.46	4.96 ± 1.12	
Overweight (25–30 kg/m^2^)	23.87	4.84 ± 1.13	
Obesity class I (30–35 kg/m^2^)	7.06	4.66 ± 1.14	
Obesity class II (35–40 kg/m^2^)	2.08	4.49 ± 1.20	
Obesity class III (≥40 kg/m^2^)	0.88	4.43 ± 1.12	
**BMI (kg/m^2^)^d^**	24.13 ± 4.53	−0.09 (−0.10, −0.08)	<0.0001
**Diet quality (mPNNS-GS)^e^**	7.61 ± 1.46	0.01 (0, 0.02)	0.0480
**Food group consumption (g/d)^f^**			
Fruit and vegetables	455.36 ± 223.86	0 (−0.01, 0.01)	0.87
Seafood	36.03 ± 30.79	0.01 (−0.01, 0.02)	0.33
Meat and poultry	71.08 ± 43.18	−0.05 (−0.06, −0.03)	<0.0001
Processed meat			0.13
No intake	16.97	4.92 ± 1.17	
Low intake	33.01	4.88 ± 1.13	
High intake	50.02	4.90 ± 1.12	
Eggs			0.0049
No intake	19.52	4.91 ± 1.15	
Low intake	31.64	4.93 ± 1.11	
High intake	48.84	4.87 ± 1.14	
Dairy products	144.01 ± 133.35	−0.04 (−0.05, −0.02)	<0.0001
Cheese	34.50 ± 24.62	0.01 (0, 0.02)	0.17
Milk-based desserts			0.0079
No intake	29.53	4.92 ± 1.15	
Low intake	20.47	4.92 ± 1.11	
High intake	50.00	4.87 ± 1.13	
Starchy foods	219.57 ± 86.45	0.02 (0, 0.03)	0.0076
Wholegrain foods	38.37 ± 43.60	0.04 (0.02, 0.05)	<0.0001
Legumes			0.042
No intake	42.45	4.87 ± 1.15	
Low intake	7.54	4.91 ± 1.13	
High intake	50.01	4.91 ± 1.12	
Fats	21.66 ± 13.06	0.04 (0.02, 0.05)	<0.0001
Sugary and fatty foods	75.5 ± 52.08	−0.02 (−0.03, −0.01)	0.0022
Sugar and confectionery	29.08 ± 25.88	0.01 (−0.01, 0.02)	0.23
Fast food			<0.0001
No intake	22.41	4.84 ± 1.18	
Low intake	27.58	4.87 ± 1.14	
High intake	50.01	4.94 ± 1.10	
Appetizers			<0.0001
No intake	33.08	4.85 ± 1.17	
Low intake	16.91	4.87 ± 1.12	
High intake	50.01	4.94 ± 1.10	
Non-salted oleaginous fruits			<0.0001
No intake	38.07	4.85 ± 1.14	
Low intake	11.65	4.90 ± 1.11	
High intake	50.28	4.93 ± 1.13	
Sweetened beverages			0.28
No intake	27.95	4.89 ± 1.13	
Low intake	22.10	4.88 ± 1.14	
High intake	49.95	4.91 ± 1.13	
Alcoholic beverages			<0.0001
No intake	20.64	4.80 ± 1.2	
Low intake	29.36	4.85 ± 1.14	
High intake	50.00	4.97 ± 1.09	
**Snacking^g^**			<0.0001
No	13.73	4.98 ± 1.19	
Yes	86.27	4.87 ± 1.13	
**Snacking frequency^g^**			<0.0001
Never	13.73	4.98 ± 1.19	
< Once a week	23.13	4.95 ± 1.11	
≥Once a week (< once a day)	43.85	4.90 ± 1.11	
≥Once a day	19.29	4.71 ± 1.19	
**Eating disorder symptoms (SCOFF)^c^**			<0.0001
No	85.39	4.95 ± 1.12	
Yes	14.61	4.51 ± 1.19	
**Category of eating disorders (SCOFF)^h^, ^i^**			<0.0001
No eating disorders	85.39	4.95 ± 1.12	
Restrictive disorders	1.01	4.49 ± 1.29	
Bulimic disorders	3.73	4.54 ± 1.21	
Hyperphagic disorders	7.89	4.47 ± 1.17	
Other eating disorders	1.98	4.61 ± 1.22	

a *% for categorical variables/non-normally distributed intake of food groups, Mean ± SD for continuous variables/normally distributed intake of food groups.*

b *PMS, Pearlin Mastery Scale; Spearman correlation coefficients (with 95 % CI).*

c *P-values based on Spearman correlation coefficients for continuous variables and Student t-test or ANOVA for categorical variables.*

d *Based on n = 30,339 participants who reported anthropometric data.*

e *mPNNS-GS = Modified French National Nutrition and Health Program Guideline Score. Based on n = 25,024 participants who had available data to calculate diet quality.*

f *G/d = Gram per day. Based on n = 22,209 participants who completed ≥3 dietary records.*

g *Based on n = 30,620 participants who completed the snacking assessment.*

h *SCOFF = Sick-Control-One-Fat-Food Questionnaire. Based on n = 28,951 participants who completed the SCOFF.*

i *Classification of ED categories based on the Expali algorithm (46)*.

### Psychometric Properties of the PMS

In the overall sample (*N* = 32,588), McDonald‘s ω was 0.842, 95% CI [0.839, 0.845], supporting the reliability of the PMS. The CFA with mastery as the single common factor provided the following fit indices: CFI = 0.917, SRMR = 0.058, RMSEA = 0.126, 90% CI [0.124; 0.129], χ^2^ (14, *N* = 32,588) = 7273.454, *p* < 0.0001. This indicates a good value of SRMR and an adequate value of CFI, but an unsatisfactory value of RMSEA. Except for item 6 that showed a satisfactory factor loading (0.40), all other items showed high factor loadings (0.50–0.84).

### Relationship Between Mastery and Weight Status

Table 3 shows the results of the logistic regression models between mastery and BMI categories. In the adjusted model, females with a higher level of mastery were less likely to be underweight, overweight or obese (all classes) than females with a lower level of mastery. ORs were the lowest in females with obesity, in particular obesity class II and III. In the adjusted model, males with a higher level of mastery were less likely to be obese (class III) than males with a lower level of obesity. There was no significant association between mastery and underweight, overweight, obesity class I and II. An additional model was tested with depressive symptomatology taken into account as a confounder. In these sensitivity analyses, results were similar in females (all *p* < 0.05), while in males, the association between mastery and obesity (class III) was no longer significant (*p* = 0.11).

**Table 3:** Association between mastery (PMS) and weight status (BMI categories) in *n* = 30,339 participants, stratified by sex (NutriNet-Santé cohort study, 2014).

	**Unadjusted model**		**Adjusted model^a^**	
	**OR (95% CI)**	***P* ^b^**	**OR (95% CI)**	***P* ^b^**
**Females^c^**				
Underweight	0.90 (0.86, 0.95)^d^	<0.0001	0.88 (0.84, 0.93)	<0.0001
Normal weight	Ref	Ref
Overweight	0.87 (0.85, 0.90)	<0.0001	0.94 (0.91, 0.97)	<0.0001
Obesity class I	0.78 (0.74, 0.81)	<0.0001	0.86 (0.82, 0.90)	<0.0001
Obesity class II	0.68 (0.63, 0.73)	<0.0001	0.76 (0.71, 0.82)	<0.0001
Obesity class III	0.69 (0.62, 0.76)	<0.0001	0.77 (0.69, 0.86)	<0.0001
**Males^e^**				
Underweight	0.95 (0.76, 1.20)	0.67	1.00 (0.79, 1.26)	0.97
Normal weight	Ref	Ref
Overweight	0.95 (0.91, 1.00)	0.047	1.01 (0.96, 1.06)	0.69
Obesity class I	0.84 (0.78, 0.91)	<0.0001	0.94 (0.86, 1.02)	0.15
Obesity class II	0.96 (0.80, 1.16)	0.69	1.10 (0.91, 1.33)	0.33
Obesity class III	0.67 (0.51, 0.87)	0.0030	0.75 (0.57, 0.99)	0.045

*PMS, Pearlin Mastery Scale.*

a *Model adjusted for age, educational level, occupational status, monthly household income, energy intake, smoking and physical activity.*

b *P-values based on multinominal logistic regressions, with mastery as a continuous independent variable.*

c *Based on n = 23,471 females who reported anthropometric data.*

d *For example, an increase of one-point in mastery is associated with a decrease in the odds (OR = 0.90 [95%CI: 0.86–0.95)] of being underweight (compared with normal weight) (all such values).*

e *Based on n = 6,868 males who reported anthropometric data*.

### Relationship Between Mastery and Diet Quality as Well as Food Group Consumption

Table 4 presents the results of the linear regression models between mastery and diet quality as well as normally distributed intake of food groups. In the adjusted model, mastery was positively associated with diet quality and with consumption of fruit and vegetables, seafood, and wholegrain foods. Mastery was associated with a lower consumption of meat and poultry, dairy products, and sugary and fatty products. No association was found with cheese, starchy foods, fats, and sugar and confectionery. Table 5 presents the results of the logistic regression models between mastery and non-normally distributed intake of food groups. In the adjusted model, participants with a higher level of mastery were more likely to have a high intake of legumes, non-salted oleaginous fruits and a low or high intake of alcoholic beverages than participants with a lower level of mastery. In addition, they were less likely to have a high intake of milk-based desserts and sweetened beverages than participants with a lower level of mastery. No association was found with processed meat, eggs, fast food and appetizers. The sensitivity analyses showed similar results, except for an absence of association between mastery and the consumption of fruit and vegetables (*p* = 0.11).

**Table 4:** Associations between mastery (PMS), diet quality and consumption of food groups normally distributed (NutriNet-Santé cohort study, 2014).

	**Unadjusted model**		**Adjusted model^a^**	
	**Beta-coefficient** **(95% CI)**	***P* ^b^**	**Beta-coefficient** **(95% CI)**	***P* ^b^**
**Diet quality (mPNNS-GS)^c^**	0.003 (0.001, 0.005)	0.0102	0.033 (0.018, 0.048)	<0.0001
**Food group consumption (g/d)^d^**				
Fruit and vegetables	1.83 (−0.77, 4.43)^5^	0.17	5.48 (3.00, 7.96)	<0.0001
Seafood	0.28 (−0.08, 0.64)	0.13	0.5 (0.14, 0.85)	0.0058
Meat and poultry	−1.47 (−1.97, −0.97)	<0.0001	−1.01 (−1.5, −0.51)	<0.0001
Dairy products	−3.94 (−5.49, −2.39)	<0.0001	−3.51 (−5.09, −1.93)	<0.0001
Cheese	0.29 (0.01, 0.58)	0.047	0.03 (−0.24, 0.31)	0.82
Starchy foods	1.62 (0.61, 2.62)	0.0016	0.18 (−0.71, 1.06)	0.70
Wholegrain foods	1.76 (1.25, 2.28)	<0.0001	1.64 (1.13, 2.15)	<0.0001
Fats	−0.16 (−0.31, −0.01)	0.045	0.1 (−0.05, 0.25)	0.20
Sugary and fatty foods	−0.01 (−0.62, 0.60)	0.97	−1.69 (−2.25, −1.13)	<0.0001
Sugar and confectionery	−0.03 (−0.33, 0.27)	0.85	0.03 (−0.26, 0.33)	0.83

*PMS, Pearlin Mastery Scale.*

a *Model adjusted for age, sex, educational level, occupational status, monthly household income, energy intake, smoking, physical activity and number of 24-h dietary records.*

b *P-values based on linear regressions, with mastery as a continuous independent variable.*

c *mPNNS-GS = Modified French National Nutrition and Health Program Guideline Score. Based on n = 25,024 participants who had available data to calculate diet quality.*

d *G/d = Gram per day. Based on n = 22,209 participants who completed ≥3 24 h dietary records.*

*^e^For example, a one-point increase in mastery was associated with an increase of 1.83 G/d (−0.77, 4.43) of fruit and vegetable intake (all such values)*.

**Table 5:** Associations between mastery (PMS) and consumption of food groups non-normally distributed (NutriNet-Santé cohort study, 2014).

	**Unadjusted model**		**Adjusted model^a^**	
	**OR (95% CI)**	***P* ^b^**	**OR (95% CI)**	***P* ^b^**
**Food group consumption^c^**				
**Processed meat**				
No intake	Ref	Ref
Low intake	0.97 (0.93, 1.00)^4^	0.053	0.98 (0.94, 1.01)	0.21
High intake	0.98 (0.95, 1.02)	0.34	0.98 (0.95, 1.02)	0.27
**Eggs**				
No intake	Ref	Ref
Low intake	1.01 (0.98, 1.05)	0.51	1.01 (0.97, 1.04)	0.79
High intake	0.97 (0.94, 1.00)	0.052	0.99 (0.96, 1.03)	0.73
**Legumes**				
No intake	Ref	Ref
Low intake	1.03 (0.98, 1.08)	0.22	1.04 (0.99, 1.09)	0.15
High intake	1.03 (1.01, 1.06)	0.014	1.04 (1.01, 1.07)	0.0043
**Fast food**				
No intake	Ref	Ref
Low intake	1.02 (0.99, 1.05)	0.29	1.01 (0.98, 1.05)	0.44
High intake	1.08 (1.05, 1.11)	<0.0001	1.01 (0.98, 1.05)	0.40
**Milk-based desserts**				
No intake	Ref	Ref
Low intake	0.99 (0.96, 1.03)	0.71	0.99 (0.96, 1.03)	0.78
High intake	0.96 (0.94, 0.99)	0.0044	0.95 (0.92, 0.98)	0.0007
**Non-salted oleaginous fruits**				
No intake	Ref	Ref
Low intake	1.04 (1.00, 1.08)	0.075	1.03 (0.99, 1.08)	0.11
High intake	1.06 (1.04, 1.09)	<0.0001	1.08 (1.05, 1.11)	<0.0001
**Appetizers**				
No intake	Ref	Ref
Low intake	1.02 (0.99, 1.06)	0.26	1.01 (0.97, 1.05)	0.62
High intake	1.08 (1.05, 1.10)	<0.0001	1.02 (0.99, 1.05)	0.17
**Sweetened beverages**				
No intake	Ref	Ref
Low intake	0.99 (0.96, 1.02)	0.51	0.98 (0.94, 1.01)	0.16
High intake	1.01 (0.98, 1.04)	0.38	0.95 (0.92, 0.98)	0.0006
**Alcoholic beverages**				
No intake	Ref	Ref
Low intake	1.04 (1.01, 1.08)	0.017	1.06 (1.02, 1.10)	0.0016
High intake	1.14 (1.11, 1.18)	<0.0001	1.12 (1.08, 1.15)	<0.0001

*PMS, Pearlin Mastery Scale.*

a *Model adjusted for age, sex, educational level, occupational status, monthly household income, energy intake, smoking, physical activity and number of 24-h dietary records.*

b *P-values based on multinomial logistic regressions, with mastery as a continuous independent variable.*

c *Based on n = 22,209 participants who completed ≥3 24 h dietary records. No intake; low intake < median intake; high intake ≥ median intake.*

*^d^For example, an increase of one-point is associated with a decrease in the odds [OR = 0.97 (95%CI: 0.93, 1.00)] of having a low intake of processed meat (compared with no intake) (all such values)*.

### Relationship Between Mastery and Snacking

Table 6 presents the results of the logistic regression models between mastery and snacking. In the adjusted model, participants with a higher level of mastery were less likely to snack than participants with a lower level of mastery. The ORs decreased with higher snacking frequency. Overall, the sensitivity analyses showed equivalent results with the main observations (all *p* < 0.05).

**Table 6:** Association between mastery (PMS) and snacking (NutriNet-Santé cohort study, 2014).

	**Unadjusted model**		**Adjusted model^a^**	
	**OR (95% CI)**	***P* ^b^**	**OR (95% CI)**	***P* ^b^**
**Overall snacking^c^**				
No	Ref		Ref	
Yes	0.92 (0.89, 0.94)^d^	<0.0001	0.89 (0.86, 0.91)	<0.0001
**Snacking frequency^c^**				
Never	Ref		Ref	
< Once a week	0.98 (0.94, 1.01)	0.18	0.95 (0.92, 0.99)	0.0095
≥Once a week (< once a day)	0.94 (0.91, 0.97)	<0.0001	0.89 (0.86, 0.92)	<0.0001
≥Once a day	0.81 (0.79, 0.84)	<0.0001	0.79 (0.76, 0.82)	<0.0001

*PMS, Pearlin Mastery Scale.*

a *Model adjusted for age, sex, educational level, occupational status, monthly household income, energy intake, smoking, and physical activity.*

b *P-values based on binary (no vs. yes) and multinominal (snacking frequency) logistic regressions, with mastery as a continuous independent variable.*

c *Based on n = 30,620 participants who completed the snacking assessment.*

d *For example, an increase of one-point is associated with a decrease in the odds [OR = 0.92 (95%CI: 0.89–0.94)] of snacking (compared with no snacking) (all such values)*.

### Relationship Between Mastery and ED Symptoms

Table 7 presents the results of the logistic regression models between mastery and ED symptoms. In the adjusted model, participants with a higher level of mastery were less likely to have ED symptoms (restrictive/bulimic/hyperphagic and other EDs) than participants with a lower level of mastery. Overall, the sensitivity analyses showed equivalent results with the main observations (all *p* < 0.01).

**Table 7:** Association between mastery (PMS) and eating disorder symptoms (NutriNet-Santé cohort study, 2014).

	**Unadjusted model**		**Adjusted model^a^**	
	**OR (95% CI)**	***P* ^b^**	**OR (95% CI)**	***P* ^b^**
**Eating disorder symptoms (SCOFF)^c^**				
No	Ref		Ref	
Yes	0.72 (0.70, 0.74)^d^	<0.0001	0.73 (0.71, 0.75)	<0.0001
**Category of eating disorders (SCOFF)^c^, ^e^**				
No eating disorders	Ref		Ref	
Restrictive disorders	0.71 (0.65, 0.78)	<0.0001	0.67 (0.61, 0.74)	<0.0001
Bulimic disorders	0.73 (0.70, 0.77)	<0.0001	0.69 (0.66, 0.73)	<0.0001
Hyperphagic disorders	0.70 (0.68, 0.73)	<0.0001	0.73 (0.71, 0.76)	<0.0001
Other eating disorders	0.77 (0.72, 0.83)	<0.0001	0.79 (0.74, 0.85)	<0.0001

*PMS, Pearlin Mastery Scale.*

a *Model adjusted for age, sex, educational level, occupational status, monthly household income, energy intake, smoking, and physical activity.*

b *P-values based on binary (no vs. yes) and multinominal (categories of eating disorders) logistic regressions, with mastery as a continuous independent variable.*

c *SCOFF, Sick-Control-One-Fat-Food Questionnaire. Based on n = 28,951 participants who completed the SCOFF.*

d *For example, an increase of one-point in mastery is associated with a decrease in the odds [OR = 0.72 (95%CI: 0.70–0.74)] of having eating disorder symptoms (compared with no eating disorder symptoms) (all such values).*

e *Classification of eating disorders categories based on the Expali algorithm (46)*.

## Discussion

The aim of this study was to investigate the association between level of mastery and weight status, food intake, snacking and EDs in a large sample of French adults. We found that females with a higher level of mastery were less likely to be underweight, overweight or obese (all classes) than females with a lower level of mastery and that males with a higher level of mastery were less likely to be obese (class III) than males with a lower level of mastery. In addition, mastery was associated with better diet quality overall, a higher consumption of fruit and vegetables, seafood, wholegrain foods, legumes, non-salted oleaginous fruits, and alcoholic beverages and with a lower consumption of meat and poultry, dairy products, sugary and fatty products, milk-based desserts and sweetened beverages. Furthermore, mastery was associated with a lower snacking frequency and less ED symptoms.

### Level of Mastery According to Sociodemographic and Lifestyle Characteristics

The mean overall mastery score in our study was comparable to previous studies (10, 53). In line with the literature, mastery was higher in males (10, 11, 19), in participants who were younger (10, 24), had a higher education (54), income (55), physical activity (10, 21), no depressive symptomatology (13, 15, 19) and were self-employed/farmers or managerial staff/intellectual professions (53). Current smokers showed the highest level of mastery. This relationship has to be further examined since data in the literature are contradictory (10, 21, 24).

### Relationship Between Mastery and Weight Status

Our results showing that females with a higher level of mastery were less likely to be underweight, overweight or obese (all classes) were maintained when controlling for depressive symptomatology and are in line with some studies (10, 15, 27), but not others (18–20). Since mastery goes along with beliefs about the general controllability of the environment (56), individuals with a high level of mastery might perceive their life circumstances as a result of their own behavior and choices. This may lead to more health-promoting behaviors (56) and thus to a more favorable weight status. Mastery could also buffer the negative effect of stress on weight gain as suggested by another study (27). Our results showed only limited associations between mastery and weight status in males which is reflected in inconsistent results from previous studies (10, 15, 18, 19). These sex-specific associations might be explained by lower prevalence rates of perceived weight discrimination in males and by the fact that males with obesity are generally more accepted than females with obesity (57, 58). Mastery might be required when being judged by others and might therefore play a more important role in females than in males regarding weight gain. Given that our data are cross-sectional, the causal relationship between mastery and weight status remains unclear. Reciprocal links are conceivable, e.g., in the course of an intervention promoting obesity-provoking behavior, mastery decreased (59). Further studies on the mutual influence and dynamics between mastery and weight status are needed.

### Relationship Between Mastery and Diet Quality as Well as Food Group Consumption

In line with a previous study in a representative sample (21), our results showed that mastery was associated with better diet quality overall in both males and females and more specifically, with a higher consumption of several healthy food groups (e.g., wholegrain foods) and a lower consumption of several unhealthy food groups (e.g., sugary and fatty foods). However, it has been proposed that the perceived utility from healthy eating might be sex-specific which might influence the relationship between mastery and healthy food choices (21). Males with a high level of mastery expected higher returns (a better health status) of their health-promoting behaviors while females with a high level of mastery showed more health-promoting behaviors as they may derive more pleasure out of these behaviors (21). Other studies showed no association with DASH (dietary approaches to stop hypertension) adherence (22) or specific food groups in specific populations with small sample sizes (23, 24). Although mastery was mainly associated with a healthier food intake, we also found that mastery was associated with a higher intake of alcoholic beverages, in line with previous studies (21, 60). Social support is positively correlated with level of mastery (60), which might lead to a wider social circle and an increased opportunity to share convivial meals, during which alcoholic beverages are often consumed (61, 62). Further, the perception of having control over life circumstances might also lead to the perception of having better coping strategies to deal with the effects of alcohol (21). This might explain why mastery is associated with a healthier food intake overall, but also with a higher consumption of alcoholic beverages.

### Relationship Between Mastery and Snacking

To the best of our knowledge, this is the first study investigating the relationship between mastery and snacking. Individuals with a higher level of mastery were less likely to snack, and the ORs decreased with snacking frequency. This is in line with the idea that a high level of mastery is related to more deliberate choices and less affective choices (21). As we live in an obesogenic environment where snacks, especially high-calorie snacks, are available almost anytime, a global sense of controllability and autonomy might go along with the capacity of resisting these temptations. In contrast, when individuals experience feelings of helplessness and desperation, one maladaptive coping strategy might be to snack in order to seek comfort (emotional eating) (63). However, more research about potential mechanisms between mastery and snacking is needed.

### Relationship Between Mastery and ED Symptoms

In line with some studies (19, 27), but not all (20), individuals with a higher level of mastery were less likely to have ED symptoms than individuals with a lower level of mastery. A low level of mastery is reflected by feelings of helplessness and feelings of being exposed to stressors without having adequate coping strategies. Thus, a low level of mastery might contribute to the development of an ED and the key features of EDs, e.g., constantly monitoring eating behavior, body weight and shape might represent an attempt to gain back a sense of control (64). However, as our results were only based on cross-sectional data, we cannot draw any conclusions about the causal relationship between mastery and EDs. It is also conceivable that experiences as loss of control eating or purging might lead to a lower level of mastery.

### Techniques to Enhance Mastery

As results have shown that mastery is associated with favorable outcomes overall, it may be a potential facilitator in promoting healthy dietary behavior. Mastery can be enhanced with cognitive techniques by increasing the understanding of the relevance of cognitive control in daily life (65). Tools to enhance mastery can be examining personal choices, planning desirable daily activities, coping with problems that cannot be changed and promoting a more satisfying lifestyle (65).

### Strengths and Limitations

One strength of the study is the large population-based sample that allowed taking into account various confounders. However, the fact that the participants were recruited on a voluntarily basis could imply a strong interest in health and nutrition topics. Thus, a selection bias cannot be ruled out (66, 67). To our knowledge, this is the first study investigating the association between mastery and food group consumption based on ≥3 24-h dietary records that serve as a good indicator of usual diet. Further, we conducted sensitivity analyses by controlling for depressive symptomatology and results were comparable to the main observations. The SCOFF was used to assess ED symptoms. Due to its good sensitivity and specificity it is recommended as a screening tool (41–43). However, we observed a low reliability of the scale in line with other studies (68, 69), which can be explained by its heterogenous items. We used the Expali algorithm, which enables distinguishing among the main categories of EDs. Nevertheless, the SCOFF cannot substitute for a clinical diagnosis, and we cannot exclude the possibility of having a certain number of false positive or false negative responses. The web-based self-report of height and weight could have led to random and systematic errors (70). However, standardized clinical measurements in a subsample (*N* = 2,513) of the NutriNet-Santé study showed good convergence with self-reported data (71) and the large sample size can contribute to a minimization of the impact of measurement error (72). Our data supported the reliability of the PMS. However, although the indices of CFI and SRMR were adequate, RMSEA exceeded recommended cut-off values (52, 73). Still, the inconsistency of CFI and RMSEA does not necessarily have to result in a rejection of the model (74). Another limitation is the cross-sectional design. As previous studies have shown that mastery varies over time and is influenced by critical life experiences (75), causal studies are needed. Finally, our paper focused on mastery as one aspect of control. However, there is a lack of consensus on theories and definitions with regard to control-related constructs, e.g., self-efficacy or locus of control (76). This might lead to difficulties in comparing and interpreting results.

## Conclusions

Our results showed that females with a higher level of mastery were less likely to be underweight, overweight, or obese (all classes) while associations were more limited in males as males with a higher level of mastery were only less likely to be obese (class III). Mastery was associated with a better diet quality overall and with a higher consumption of several healthy food groups, e.g., wholegrain foods as well as with a lower consumption of several unhealthy food groups, e.g., sugary and fatty foods. However, mastery was also associated with a higher intake of alcoholic beverages. In addition, individuals with a higher level of mastery were less likely to snack and to have ED symptoms. As our results revealed that mastery is associated with favorable outcomes, mastery may be a potential facilitator in promoting healthy dietary behavior. However, research based on longitudinal designs and randomized-controlled trials are needed to further investigate these associations.

## Data Availability Statement

The datasets presented in this article are not readily available because the data of this study are protected under the protection of health data regulation set by the Commission Nationale de l'Informatique et des Libertés (CNIL). If you are a researcher of a public institution, you can submit a collaboration request including your institution and a brief description of your project to collaboration@etude-nutrinet-sante.fr. All requests will be reviewed by the steering committee of the NutriNet-Santé study. A financial contribution may be requested. If the collaboration is accepted, a data access agreement will be necessary and appropriate authorizations from the competent administrative authorities may be needed. In accordance with existing regulations, no personal data will be accessible. Requests to access the datasets should be directed to collaboration@etude-nutrinet-sante.

## Ethics Statement

The NutriNet-Santé cohort study was conducted in line with the guidelines of the Declaration of Helsinki. Study procedures were approved by the Institutional Research Board of the French Institute for Health and Medical Research (IRB INSERM no. 0000388FWA00005831) and the Commission Nationale de l'Informatique et des Libertés (CNIL nos. 908450 and 909216). Electronic informed consent was given by all participants. The study was registered at clinicaltrials.gov (NCT03335644). The patients/participants provided their written informed consent to participate in this study.

## Author Contributions

UG, SP, and FE designed research (project conception, development of overall research plan, and study oversight). SP, VA, SH, and MT conducted research (hands-on conduct of the experiments and data collection). MR and UG analyzed data or performed statistical analysis. UG wrote paper. SP had primary responsibility for final content. UG, MR, NB, AN, FE, ST, VA, MT, and SP read and revised the manuscript and approved the final version. All authors contributed to the article and approved the submitted version.

## Funding

The NutriNet-Santé cohort study was supported by the following public institutions: Ministère de la Santé, Santé Publique France, Institut National de la Santé et de la Recherche Médicale (INSERM), Institut National de Recherche pour l'Agriculture, l'Alimentation et l'Environnement (INRAE), Conservatoire National des Arts et Métiers (CNAM), and Sorbonne Paris Nord Université. This research was part of the FOODPOL project, which was supported by the INRAE in the context of the 2013–2017 Metaprogramme Diet impacts and determinants: Interactions and Transitions. In addition, the project was part of the PSYCHALIM project supported by the INRAE and the Centre national de la recherche scientifique (CRNS), in the context of the 2019–2021 Défi Mutations Alimentaires. UAG was supported by NutriAct – Competence Cluster Nutrition Research Berlin- Potsdam funded by the German Federal Ministry of Education and Research (FKZ: 01EA1408A-G).

## Conflict of Interest

The authors declare that the research was conducted in the absence of any commercial or financial relationships that could be construed as a potential conflict of interest.

## Publisher's Note

All claims expressed in this article are solely those of the authors and do not necessarily represent those of their affiliated organizations, or those of the publisher, the editors and the reviewers. Any product that may be evaluated in this article, or claim that may be made by its manufacturer, is not guaranteed or endorsed by the publisher.

## Abbreviations

CES-D: Center for Epidemiological Studies-Depression
DV: dependent variable
IV: independent variable
mPNNS-GS: Modified French National Nutrition and Health Program Guideline Score
PMS: Pearlin Mastery Scale
SCOFF: Sick-Control-One-Fat-Food Questionnaire
ED: Eating disorders
CFI: Comparative fit index
RMSEA: Root mean square error of approximation
and SRMR: Standardized root mean square residual.
