# Supplementary material for: Mastery Is Associated With Weight Status, Food Intake, Snacking, and Eating Disorder Symptoms in the NutriNet-Santé Cohort Study
Source: Front Nutr. 2022 May 25;9:871669. doi: 10.3389/fnut.2022.871669 (PMC9174750; doi:10.3389/fnut.2022.871669)
Supplement: Supplementary file 1 [file Table_1.DOCX]

**Online Supporting Material**

Supplemental Figure 1: Participant flow chart from the NutriNet-Santé Cohort Study included in current analyses.

139,420 participants included in the NutriNet-Santé study

(May - November 2014)

106,403 participants who did not complete the PMS were excluded

33,017 participants completed the PMS

148 participants with an acquiescence bias were excluded

281 pregnant women were excluded

32,588 remaining participants who completed the PMS

22,209 participants completed at least three dietary records

25,024 participants had available data to calculate the mPNNS-GS

30,620 participants completed the snacking assessment

30,339 participants reported anthropome-tric data

28,951 participants completed the SCOFF
